# Analysis of Thermal Properties of Materials Used to Insulate External Walls

**DOI:** 10.3390/ma17194718

**Published:** 2024-09-26

**Authors:** Marta Pomada, Klaudia Kieruzel, Adam Ujma, Paweł Palutkiewicz, Tomasz Walasek, Janina Adamus

**Affiliations:** 1 Faculty of Civil Engineering, Czestochowa University of Technology, 69 Dabrowskiego St., 42-201 Czestochowa, Poland; marta.pomada@pcz.pl (M.P.); klaudia3112@onet.pl (K.K.); 2 Faculty of Technical Sciences, University of Applied Sciences in Nysa, 7 Armii Krajowej St., 48-300 Nysa, Poland; adam.ujma@pans.nysa.pl; 3 Faculty of Mechanical Engineering, Czestochowa University of Technology, 69 Dabrowskiego St., 42-201 Czestochowa, Poland; pawel.palutkiewicz@pcz.pl (P.P.); tomasz.walasek@pcz.pl (T.W.)

**Keywords:** building materials, thermal properties, thermal insulation, building envelope, FEM analysis, experimental tests

## Abstract

This article emphasizes the significance of understanding the actual thermal properties of thermal insulation materials, which are crucial for avoiding errors in building design and estimating heat losses within the energy balance. The aim of this study was to analyse the thermal parameters of selected thermal insulation materials, particularly in the context of their stability after a period of storage under specific conditions. The materials chosen for this study include commonly used construction insulations such as polystyrene and mineral wool, as well as modern options like rigid foam composites. Experimental studies were conducted, including the determination of the thermal conductivity coefficient *λ*, as well as numerical analyses and analytical calculations of heat flow through a double-layer external wall with a window. The numerical analyses were performed using the TRISCO software version 12.0w, based on the finite element method (FEM). A macrostructural analysis of the investigated materials was also performed. The findings indicated that improper storage conditions adversely affect the thermal properties of insulation materials. Specifically, storing materials outdoors led to a deterioration in insulating properties, with an average reduction of about 4% for the standard materials and as much as 19% for the tested composite material. Insufficient understanding of the true thermal properties of insulation materials can result in incorrect insulation layer thickness, degrading the fundamental thermal parameters of external walls. This, in turn, increases heat loss through major building surfaces, raises heating costs, and indirectly contributes to greenhouse gas emissions.

## 1. Introduction

The heating and cooling of buildings has emerged as a key issue for improving their energy efficiency, as evidenced by the findings of the report [1], which states that the buildings and construction sector significantly contributes to global climate change, accounting for approximately 21% of global greenhouse gas emissions. According to the report, in 2022, buildings constituted 34% of global energy consumption and nearly 37% of total CO_2_ emissions. In view of the challenges posed by the depletion of natural energy resources, the building envelope—the structural element of a building that separates its interior from the external environment—is becoming increasingly important. It is responsible for minimizing heat loss in the building, protecting against moisture, ensuring thermal comfort, and providing aesthetic value. The building envelope includes components such as walls, roof, windows, doors, and ground floor. As indicated in [2], windows are one of the main causes of heat loss in buildings. The specific heat flux through double-layer glazing is approximately five times greater than that through walls. Nevertheless, heat loss through walls remains significant, given that the glazing of a typical building, according to current trends, covers almost 30–50% of the wall surface. Therefore, improving the external insulation of walls is crucial to achieving energy-efficient buildings. As stated in [3,4], the building envelope accounts for 50–60% of the total heat transfer. It is primarily responsible for minimizing heat loss from inside the building to the outside. Effective thermal insulation of external walls can enhance the building’s energy efficiency, resulting in lower heating and cooling costs and ensuring satisfaction with the conditions of their use.

The most commonly utilized insulating materials are conventional organic substances, such as cork and cellulose, and inorganic materials, including mineral wool, calcium silicate, foam glass, and perlite. The use of so-called state-of-the-art materials, such as closed-cell foam, aerogel, transparent vacuum panels, and reflective multi-foils, or sustainable materials, like bio-insulation materials, agricultural waste, sheep wool, and recycled insulation materials, is less prevalent [5].

In works [5,6,7], comparative analyses of building insulation materials were conducted in terms of their thermal, hygroscopic, and acoustic properties, as well as their reaction to fire, environmental properties, and costs. Abu-Jdayil [6] and Villasmil W et al. [8] compared traditional insulators with the superinsulation materials, highlighting that state-of-the-art insulators allow for the use of thinner layers compared to conventional ones. Abu-Jdayil et al. [6] emphasize the need to develop insulation materials that have a reduced environmental impact and are cost-effective. These materials should leverage renewable resources and utilize waste products in the development of thermal insulation. In their analysis of the performance of building walls in different climate zones, Kumar et al. [5] found that walls constructed from materials with higher thermal resistance (i.e., low thermal conductivity materials) are more cost-effective in heating-dominated regions. Conversely, in areas where cooling is dominant, walls with relatively lower thermal resistance are preferred. However, the authors caution that well-insulated and airtight houses may increase the risk of overheating and peak cooling demand during hot summer periods in heating-dominated regions, although the total annual energy consumption decreases with better insulation. Asdrubali et al. [9] add that when selecting building thermal insulation materials, it is important to consider factors beyond their thermal properties, such as their ability to attenuate unwanted noise and minimize fire hazards. This approach ensures that thermal insulation materials not only enhance energy efficiency but also contribute to the overall safety and comfort within buildings.

Petrosyan [10] underlines the significance of determining the optimal thickness of thermal insulation and its proper placement. When determining the insulation thickness, factors such as the type of heat source, the heating device, and the characteristics of the heat and cold supply system should be carefully considered. Gori et al. [11] discuss the design criteria for improving insulation effectiveness of multi-layer walls. The authors state that, in addition to thickness, the order and arrangement of the layers also influence the insulating capacity of a multi-layer wall. This relates to the potential differentiation of moisture properties in thermal insulation materials operating under varying temperature conditions within the cross-section of a partition, and consequently, the differentiation of their thermal conductivity. In the case of double-layer walls, it is always more efficient to place the insulating layer as close to the outside as possible. This constitutes the fundamental criterion for designing effective insulating structures. 

The assessment of thermal insulation properties in construction materials is a crucial step in evaluating building energy efficiency. Studies by Wi et al. [12] indicate that the actual thermal and moisture properties of thermal insulation materials often differ from the values declared by manufacturers in product technical datasheets, particularly the thermal conductivity values of building materials—a key parameter from the perspective of building energy efficiency. The authors examined 21 thermal insulation materials commonly used in construction. By comparing the measured thermal conductivity values with those declared by the manufacturers, they identified a tendency for these values to differ by as much as 3% to 60%. In the study by Choi et al. [13], composite insulation materials were investigated, revealing that thermal resistance varied by approximately 4% to 8%. The authors also highlighted the dependence of the thermal conductivity coefficient on the thickness of the insulation material. 

One of the most important parameters determining the thermal properties of insulation materials is the thermal conductivity coefficient *λ*. 

Although innovative materials have emerged to meet the growing demands for energy efficiency and sustainability, their performance is less well-established compared to traditional insulators. To address this issue, Baldinelli et al. [14] conducted a Round Robin Test involving six European laboratories. They used hot plate devices to measure the thermal conductivity of four materials: aerogel, vacuum insulation panels, polystyrene, and birch wood fiber insulation boards. The results show that the test is effective for assessing these innovative materials, providing reliable absolute values and demonstrating good repeatability. However, designers should exercise caution, as these test methods are not yet widely available, and data for materials such as vacuum insulation panels may carry significant uncertainties. Furthermore, Assaad et al. [15] emphasize that thermal conductivity, a critical parameter for evaluating thermal insulation materials in buildings, is affected by both temperature and moisture content, particularly in the case of hygroscopic materials. In their study, the researchers investigated how moisture infiltration impacts the measurement of thermal conductivity in wet wood fiber insulation. They specifically addressed measurement errors that occur during adjustments to the test duration. By comparing the experimentally determined moisture-dependent true thermal conductivity with the values predicted by standards, Assaad et al. [15] provided valuable insights into the behavior of hygroscopic insulation materials.

As noted by the authors of [16], the key factors influencing the design of energy efficiency in buildings have evolved over the last few decades. In the past, the focus was primarily on project optimization and the selection of modern technologies. Currently, building management, including user behavior and building service systems, is also taken into consideration.

One of the key strategies for enhancing the energy efficiency of buildings is to effectively insulate the building envelope, especially the external walls. According to [17] external wall insulation can reduce the total energy load by up to 19.7%. This not only lowers energy bills but also helps to curb greenhouse gas emissions, contributing to broader environmental sustainability goals. Given the critical importance of insulation material, the following factors should be considered when selecting insulation for external walls:

- Thermal conductivity: materials with low thermal conductivity are more effective at reducing heat transfer.
- Durability: materials should withstand environmental conditions without degrading over time.
- Cost-effectiveness: the initial cost should be balanced against long-term savings.
- Environmental impact: life cycle assessment can evaluate the environmental footprint of different materials, taking into account production, use, and disposal.

This paper also emphasizes the importance of proper storage for thermal insulation materials, as research shows that some materials are particularly prone to absorbing moisture from the environment, which significantly affects their thermal conductivity.

In order to enhance the energy efficiency of buildings, numerical analyses based on the finite element method (FEM) are commonly employed, particularly for calculating heat fluxes through building envelopes. However, the substantial computational cost associated with FEM simulations often limits parametric studies and design optimization. To address this challenge, the authors of [18] propose a domain-informed finite element machine learning (ML) framework. The methodology involves the following steps: first, a set of heat flux training data is generated using FEM simulations. Then, ML is applied to predict heat fluxes for scenarios not covered by numerical calculations. Remarkably, this approach yields equally accurate results in significantly less time, reducing the analysis duration from approximately 12 h using FEM to less than 1 min with ML-assisted heat flux prediction. ML is an increasingly utilized data-driven method that combines computer science and statistics. It comes from the core of artificial intelligence and data science [19]. In contrast to physics-based models, which rely on detailed physical principles, ML models make predictions without explicitly understanding the underlying physics. These ML models are often referred to as black-box models [20]. In the context of building energy studies, ML has found widespread application in predicting various factors, including a building’s thermal load [21,22], energy consumption [23], wall heat flux [24], heat loss coefficient [25], and other related factors. 

To reduce the demand for thermal energy in buildings, enhance user comfort, and improve the impact of buildings on human health, it is essential to understand the processes occurring in the various building components and the materials from which they are made. A thermal-humidity analysis based on numerical calculations is a fundamental aspect of proper building envelope design. The aim of this study was to analyze the thermal parameters of selected thermal insulation materials, with a focus on their stability after a storage period under specific conditions. The experimental studies, which involved determining the thermal conductivity coefficient *λ*, were complemented by analytical calculations and numerical analyses of the heat flow through a double-layer wall. Numerical analyses were conducted using the FEM-based program TRISCO. 

The findings of this study have practical implications for the construction industry, providing guidelines for selecting the most effective insulation materials. This can help to bridge the gap between research and practice, fostering the adoption of energy-saving technologies.

## 2. Materials and Methods

**Figure 1:**
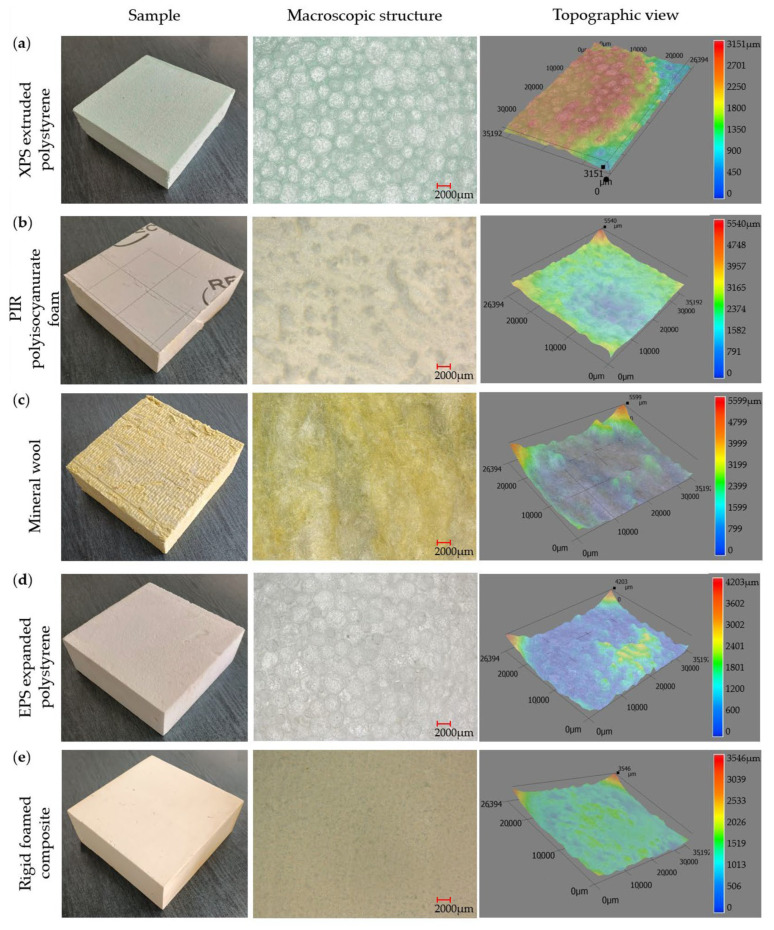
Analyzed thermal insulation materials with their macroscopic structure and topographic views: (**a**) XPS extruded polystyrene, (**b**) PIR polyisocyanurate foam, (**c**) mineral wool, (**d**) EPS expanded polystyrene, (**e**) rigid foamed composite.

The following thermal insulation materials were analyzed in this study: XPS—extruded polystyrene and EPS—expanded polystyrene, PIR—polyisocyanurate foam, mineral wool, and rigid foamed composite used in the manufacture of window mounting frames (Figure 1). The macrostructural analysis was conducted using a VHX-900F microscope (Keyence, Mechelen, Belgium), in reflected light. Surface images of the analyzed materials were captured at 10× magnification. In addition, a three-dimensional topographic representation of the sample surfaces was provided, showing the shape, roughness, and possible surface contamination.

The insulating properties of the analysed materials were determined by measuring the thermal conductivity *λ* using a heat flow meter, specifically the NETZSCH HFM 446 Lambda Medium [26]. This heat flow meter is designed for testing materials with low to medium thermal conductivity and allows testing in the temperature range from −30 °C to +90 °C.

To examine the effect of different storage conditions on the thermal conductivity coefficient *λ*, measurements were taken for samples before and after a storage period. The obtained results were compared with the thermal conductivity values declared by the manufacturers, *λ_D_*. The declared values are summarized in Table 1.

The samples for experimental testing were divided into three groups. The first group of samples served as the control group, with tests conducted immediately after receiving the material from the manufacturer. The second group consisted of samples taken from materials stored inside an unheated storage room that shielded them from direct exposure to atmospheric agents such as atmospheric precipitation and solar radiation. During the storage period, the temperature in the room ranged from 0 °C to +10 °C. The third group comprised samples of materials stored in an outdoor environment (i.e., in the open air), where they were exposed to wind, snow, rain, frost, and solar radiation. The ambient temperature during the storage period varied between −2 °C and +15 °C. Samples from both the second and third groups were seasoned for six months during the winter-spring period.

The measured values of thermal conductivity coefficients were used to analyze the impact of changes in the insulating properties of the materials on the heat flow through external walls insulated with these materials. The thermal analysis of the walls aimed to determine the following:

- the heat transfer coefficient through the external wall *U_c_*,
- the influence of linear thermal bridge on the thermal transmittance of the external wall,
- the temperature distribution within the external wall,
- the risk of condensation on the internal wall surface (risk of mold growth).

Numerical analyses were performed using the TRISCO program [27,28], a digital tool for thermal analysis of building components. The program enables steady-state thermal simulations for orthogonal/rectangular building elements in both 2D and 3D. Numerical simulations are conducted in accordance with current EN ISO standards [29,30,31] and enable the analysis of thermal bridges: calculation of heat losses, and estimation of surface condensation risk. The program allows for the application of point, surface, and volumetric boundary conditions in line with EN standards. Surface boundary conditions are applied at the material–environment boundary or between materials; volumetric boundary conditions are assigned to the material volume with a constant temperature or heat flux density and point boundary conditions are described by constant temperature or power. 

### 2.1. Experimental Research in a Plate Apparatus

**Figure 2:**
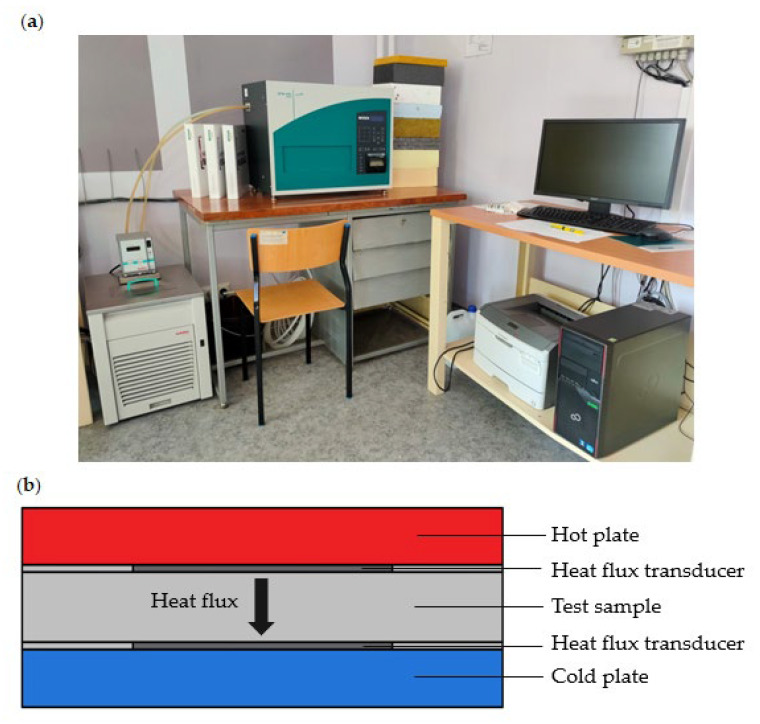
HFM 446 Lambda Medium Measurement Apparatus: (**a**) test rig, (**b**) schematic representation and operational principles of the apparatus.

The test rig for measuring the thermal conductivity coefficient *λ* is shown in Figure 2a, while the schematic representation of the plate apparatus and its operational principles are illustrated in Figure 2b.

As shown in Figure 2b, the test sample is placed between two plates, with the upper plate serving as the heating plate and the lower plate acting as the cooling plate. By recording the steady-state heat flow *Q* through the plates, the thermal conductivity *λ* of the analyzed material can be calculated using the Fourier heat flow Equation (1):

(1) $$\lambda =\frac{Q\cdot L}{A\cdot \Delta T}$$

where:

*λ*—thermal conductivity [W/(m·K)]
*Q*—heat flux [W],
*A*—sample area [m^2^],
*L*—sample thickness [m],
Δ*T*—temperature difference between the plates [K].

The two heat flux transducers (HFTs) depicted in Figure 2b measure the heat flow through the sample. The test is concluded when thermal equilibrium is reached. Additionally, plate thermocouples allow direct measurement of the temperature on the surface of the samples.

The samples for measuring thermal conductivity coefficient were of the following dimensions: 0.305 × 0.305 × 0.10 m. The tests were conducted under the following conditions:

– temperature of the cold plate: −10 °C,
– temperature of the hot plate: 10 °C,
– temperature of the sample: 23 °C,
– pressure: 2 kPa,
– air temperature around the test stand: 20 °C,
– ambient relative humidity: 50%.

The result of the test is presented as the average value from measurements of the thermal conductivity coefficient *λ_avg_*. Each sample was subjected to three tests, considering different orientations. After each test, the sample was either inverted or rotated 180°. Prior to each thermal conductivity measurement, the temperature and relative humidity of the laboratory were recorded. The dimensions of the samples were verified by measuring the edge lengths and calculating the average. Furthermore, the thickness of the samples was measured using the plate apparatus after adjusting the heating plate load. To prevent moisture from penetrating the insulation materials being tested, the edges of the samples were sealed with tape.

### 2.2. Numerical Analyzes and Analytical Calculations

The TRISCO programme, used for thermal calculations, facilitated three-dimensional modelling of heat flow, the determination of heat transfer coefficients *U*, and the assessment of the risk of condensation on the surface of the building envelope (in this case, an external wall with a window).

A series of numerical analyses and analytical calculations were conducted for various configurations of a double-layer external wall with a window mounted within the thermal insulation layer. The window installation system, which employs a specially profiled composite frame, has been extensively discussed in [27,32,33].

**Figure 3:**
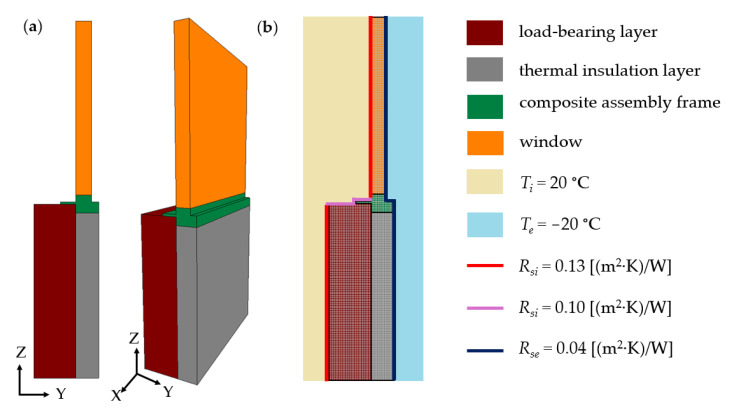
Double-layer external wall model: (**a**) computational model, (**b**) numerical model.

The TRISCO numerical model of the building envelope, comprising a double-layer wall with a window, is illustrated in Figure 3.

The external wall models were based on the following assumptions:

– a load-bearing layer, 0.24 m thick, made of cellular blocks with a thermal conductivity coefficient *λ* = 0.16 W/(m·K),
– a thermal insulation layer, 0.10 m thick, made of the five analyzed insulating materials,
– a composite mounting frame made of material with a thermal conductivity coefficient *λ* = 0.020 W/(m·K),
– a window with a heat transfer coefficient *U_w_* = 0.6 W/(m^2^·K).

The numerical simulations were conducted under steady-state heat flow conditions, with the following boundary conditions: for upward heat flux *R_si_* = 0.10 m^2^·K/W, and for horizontal heat flow—the heat transfer resistance on the internal side of the envelope *R_si_* = 0.13 (m^2^·K)/W, and *R_se_* = 0.04 (m^2^·K)/W on the external side. Constant internal and external temperatures were assumed, with *T_i_* = 20 °C and *T_e_* = −20 °C, respectively. 

The analysis of the thermo-humidity parameters of the selected external wall variants included the following:

- determination of heat loss through the flat envelope by calculating the corrected heat transfer coefficient *U_c_* [W/(m^2^·K)] in accordance with EN ISO 6946 [29];
- assessment of the risk of surface condensation (mold growth) by determining the temperature factor at the internal surface *ƒ_Rsi_* [−] in accordance with EN ISO 13788 [30];
- calculation of the linear heat transmittance coefficient *Ψ* [W/(m^2^·K)] in accordance with EN ISO 10211 [31].

The primary parameter describing the thermal quality of an envelope in terms of its insulating properties is the heat transfer coefficient *U* [W/(m^2^·K)]. This coefficient is fundamental in calculating the energy balance of buildings. The lower its value, the better the thermal insulation of the envelope, resulting in reduced heat loss through heat transfer. Therefore, when calculating the *U*-value, it is essential to take into account the construction technology of the building envelope and apply corrections for:

– leaks in the thermal insulation layer,
– mechanical fasteners penetrating the thermal insulation layer.

The corrected value of the heat transfer coefficient *U_c_* is obtained by adding the correction term Δ*U*, in accordance with the formula set forth in standard [29]:

(2) $$U_{c}=U+\Delta U=U+\Delta U_{g}+\Delta U_{f}$$

where:

*U_c_*—corrected value of heat transfer coefficient [W/(m^2^·K)],
Δ*U_g_*—correction for leaks in the thermal insulation layer [W/(m^2^·K)],
Δ*U_f_*—correction for mechanical fasteners in the thermal insulation layer [W/(m^2^·K)].

In accordance with the Regulation of the Minister of [34], the *U_c_* values for walls, roofs, floors, and ceilings, calculated according to European standards and accounting for corrections, must not exceed the *U_c_*_(*max*)_ values for all types of buildings. For external walls, assuming an indoor temperature of *T_i_* ≥ 16 °C, the heat transfer coefficient *U_c_* must meet the condition:

(3) $$U_{c}\leq U_{c(max)}=0.20\;W/(m^{2}\cdot K)$$

EN ISO 13788 [30] also requires checking the risk of surface condensation, which is otherwise referred to as the risk of mold growth on the internal surface of the envelope. To assess this, the following condition must be verified:

(4) $$f_{Rsi\;}\geq \;f_{Rsi(crit)}$$

where:

*f_Rsi_*—temperature factor at the internal surface of the envelope [−],
*f_Rsi_*_(*crit*)_—critical value of the temperature factor at the internal surface of the envelope [−].

The temperature factor *f_Rsi_* can be calculated using Equation (5) or determined through computational software.

(5) $$f_{Rsi\;}=\frac{T_{si,min}-T_{e}}{T_{i}-T_{e}}$$

where:

*f_Rsi_*—temperature factor at the internal surface of the envelope [−],
*T*_*si*,*min*_—minimum internal surface temperature according to heat flow calculations [°C],
*T_e_*—external temperature used in the calculations [°C],
*T_i_*—internal temperature used in the calculations [°C].

The critical value of the temperature factor *f_Rsi_*_(*crit*)_ can be determined in two ways:

- simplified, assuming an internal temperature of *T_i_* = 20 °C and average monthly relative air humidity in the room of *φ_i_* = 50%, in which case *f_Rsi(crit)_* = 0.72,
- precise, according to the procedure described in the EN ISO 13788 standard [30].

In the present study, the temperature *f_Rsi_* was calculated using the TRISCO program and compared with the critical value *f_Rsi_*_(*crit*)_ determined by the simplified method.

The values of the linear heat transmittance coefficient *Ψ* were determined in accordance with the Formula (6):

(6) $$\Psi =L_{2D}-\sum_{i=1}^{N_{i}}U_{i}\cdot l_{i}$$

where:

*Ψ*—linear heat transmittance coefficient [W/(m·K)],
*L*_2*D*_—linear thermal coupling coefficient, representing the total heat flux through the two-dimensional joint, with a temperature difference between environments [W/(m·K)],
*U_i_*—heat transfer coefficient of the i-th component or section of the envelope [W/(m^2^·K)],
*l_i_*—length of the component or section of the envelope corresponding to the given *U_i_* value [m].

According to the Passive House Institute [35] the requirement for a linear thermal bridge-free building for buildings of standard geometry is met when:

(7) Ψ≤ 0.01 W/(m·K)

According to EN ISO 12831 [36], the thermal calculations accounted for the variable cross-section of the external wall, and the linear heat transmittance coefficient *Ψ* was calculated for each segment with a change in the cross-section of the envelope. The calculations used the heat flux values derived from the numerical simulations conducted with the TRISCO program. 

## 3. Results and Discussion

The findings of the experimental studies and numerical analyses enabled the identification of the impact that storage methods have on the insulating properties of the materials under examination. Furthermore, it was demonstrated how the inappropriate selection of insulation layer thickness and a lack of knowledge regarding the actual thermal properties of the materials affect the primary thermal parameters of external walls.

### 3.1. Experimental Research Results

The outcomes of the thermal conductivity coefficient tests for the three groups of analyzed materials are presented in Table 2, Table 3 and Table 4. Each sample was tested three times, considering different orientations. Following each test, the sample was either inverted or rotated by 180°. Additionally, basic information about the tested products, including mass, insulation material density, and measurement duration, is provided.

The conducted tests revealed notable discrepancies in measurement durations between individual samples, even for the same insulation material. The shortest measurement times were observed for polystyrenes (approximately 83 min on average for XPS and 90 min for EPS) and mineral wool (112 min). These materials exhibited minimal variation in measurement duration. In contrast, materials with the lowest thermal conductivity, i.e., PIR polyisocyanurate foam and foamed composite, required the longest measurement times. Stabilizing the heat flow, surface temperatures of the tested samples, and measuring the thermal conductivity coefficient *λ* for these materials took, on average, about 525 and 688 min, respectively. Despite its ultimately low thermal conductivity, the composite material showed high variability in the *λ* coefficient during the tests, indicating that measurements of materials with a heterogeneous structure are subject to considerable uncertainty. 

Differences in measurement times and variations in the thermal conductivity coefficient (*λ*) for individual samples id due to the method used to measure thermal conductivity with the HFM 436 Lambda apparatus. In this method, thermal conductivity is measured using heat flux sensors, with readings taken once thermal equilibrium has been achieved at a specified temperature difference and a uniform temperature gradient across the sample.

During the initial phase of measurement, the system works to meet rough equilibrium criteria. Subsequently, the system refines these to meet the fine equilibrium criteria. These criteria are determined by the calibration and testing conditions. The measurement is considered complete when the thermal conductivity coefficient (*λ*) values from the dual heat flux sensor set show repeatability within ±0.5% over a minimum of 15 min, at which point the final results are recorded.

Accurate temperature measurements on the sample surfaces and a uniform heat flux across the sample require ideal surface conditions and a homogeneous internal structure. High thermal conductivity materials often have irregular, uneven surfaces. These materials are difficult to machine to remove any surface imperfections, which can lead to uneven thermal contact with the heating plates and heat flux sensors.

Table 5 summarizes the declared thermal conductivity coefficient values for all the analyzed thermal insulation materials, along with the results obtained from the experimental tests, taking into account the storage conditions of the samples.

The thermal conductivity coefficient values for the control samples and those stored indoors were found to be almost identical to the declared values. However, for the foamed composite, both the control sample and the indoor-stored sample exhibited *λ*-values that were, on average, 0.002 W/(m·K) worse than the declared value, representing approximately an 11% increase from the value specified in the technical data sheet. All samples stored in an outdoor environment showed an increase in the thermal conductivity coefficient. For EPS and XPS polystyrenes and PIR polyisocyanurate foam, the increase was 0.001 W/(m·K); for mineral wool, it was 0.02 W/(m·K); and for the composite material, it was as much as 0.04 W/(m·K). This resulted in a deterioration of the insulating properties, with an average reduction of 4.4% for the standard materials and as much as 19% for the tested composite material. 

The results of all experimental tests were subjected to statistical analysis. For each insulation material tested, the average thermal conductivity coefficient *λ_avg_* was calculated according to Equation (8):

(8) $$\lambda_{avg}=\frac{1}{n}\sum_{i-1}^{n}\lambda_{i}$$

where:

*λ_avg_*—average thermal conductivity coefficient for *n* tests [W/(m·K)],
*λ_i_*—individual thermal conductivity coefficient [W/(m·K)],
*n*—number of tests conducted for the given insulation material.

The corresponding standard deviation (*s_d_*) was calculated according to Equation (9):

(9) $$s_{d}=\sqrt{\frac{1}{n-1}\sum_{i=1}^{n}{\left(\lambda_{i}-\lambda_{avg}\right)}^{2}}$$

The calculated data were compiled in Table 6.

The results of the standard deviation for the tests performed indicate how much the values obtained differ from the arithmetic mean of these data. A larger standard deviation reflects greater dispersion around the mean, signifying higher variability within the data set. Conversely, a smaller standard deviation suggests that the data are more concentrated around the mean, indicating lower variability. Differences between individual readings, as well as variations in measurement times for different samples, may be attributed to factors such as the method used to measure the thermal conductivity, the irregular surface of the samples, and the internal structure of the insulation material.

For the insulation materials analyzed, within each measurement group (G1 to G3), the smallest variation in results was observed for XPS extruded polystyrene. In contrast, PIR polyurethane foam and mineral wool exhibited the largest standard deviations. The consistent measurement times and minimal variability seen in XPS polystyrene are attributed to its closed-cell structure and smooth surface. Conversely, while PIR polyurethane foam also features a closed-cell structure, is coated on both sides with layers of aluminum, paper, and polyethylene. Mineral wool, due to its open structure and disordered fiber arrangement, significantly affects the accuracy of thermal conductivity measurements.

**Figure 4:**
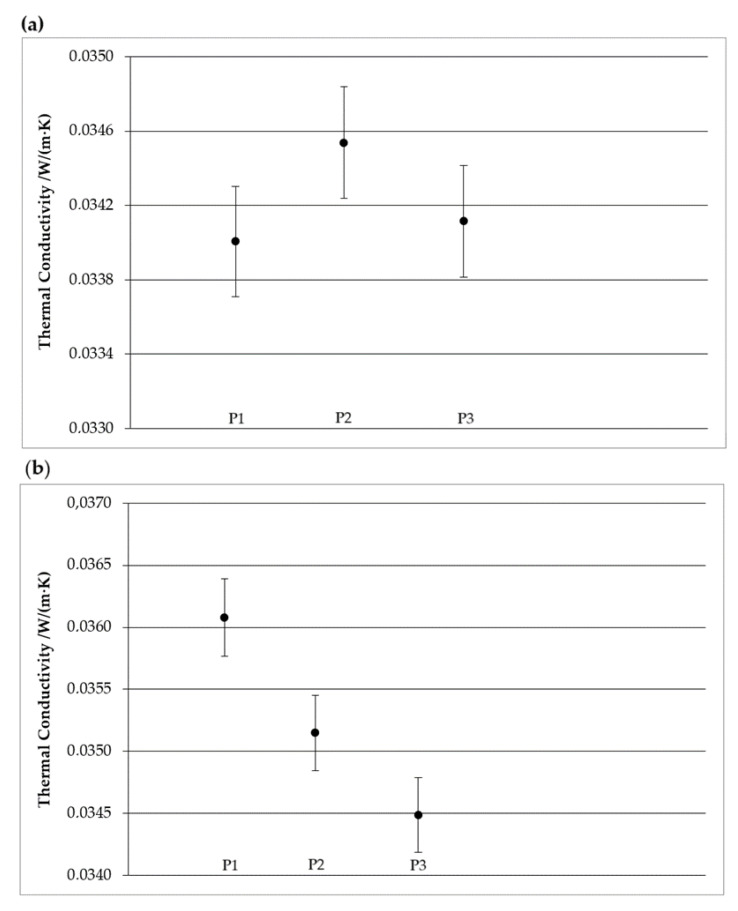
Changes in the thermal conductivity coefficient *λ* value during tests for selected samples: (**a**) XPS extruded polystyrene, (**b**) mineral wool.

Based on the test reports for the thermal conductivity coefficient *λ*, it is possible to analyze how its value changed during the testing period and within what error range its final value was calculated (e.g., report has been placed as Supplementary Materials). Figure 4a shows the *λ*-values and error bars for a control sample of XPS extruded polystyrene, while Figure 4b illustrates the same for mineral wool stored indoors. 

In the case of PIR polyisocyanurate foam and rigid foamed composite samples, difficulties were encountered during the thermal conductivity testing. The time required for temperature stabilization on the hot and cold plates was significantly extended, and in some cases, the tests had to be discontinued. 

### 3.2. Numerical Analyzes and Analytical Calculations Results

Based on the results of numerical and analytical calculations, the thermal parameters for the selected variants of external walls were analyzed. First, it was verified whether the chosen material configuration meets the thermal insulation requirements for external walls as specified in Condition (3) of the Regulation [34]. Table 7 presents the calculated values of the corrected heat transfer coefficient *U_c_* (W/m^2^·K). The calculations were conducted for a load-bearing layer made of cellular concrete blocks with a thickness of 0.24 m and a thermal conductivity coefficient of *λ* = 0.16 W/(m·K), as well as for a thermal insulating layer with a thickness of 0.10 m. The declared thermal conductivity values, *λ_D_*, for the thermal insulating materials were assumed, along with the average values, *λ_avg_*, for the samples stored outdoors.

The values obtained for the heat transfer coefficients *U_c_* demonstrate the importance of experimentally determining the thermal parameters of thermal insulating materials, especially those stored under inappropriate conditions. The *U_c_* values calculated from the experimentally measured thermal conductivity coefficients *λ_avg_* are, on average, 2.5% higher for materials 1–4 and 12% higher for the foamed composite, compared to those calculated from the declared values. This results in an underestimation of heat loss through the building envelope and impacts the overall energy balance of the building.

Table 7 highlights in red the *U_c_* values that do not meet the requirement for adequate thermal insulation of external walls. Table 8 shows how much the thermal insulation layer thickness needs to be increased to meet this requirement and provides the updated *U_c_* values.

To ensure compliance with Condition (3) for the corrected heat transfer coefficient *U_c_* of external walls, the thickness of the thermal insulation layer needs to be increased by:

- a minimum of 0.03 m to 0.04 m when using the declared thermal conductivity coefficients in the calculations,
- a minimum of 0.03 m to 0.05 m when using the measured thermal conductivity coefficients in the calculations.

The incorrect selection of thermal insulation layer thickness and the failure to account for the actual thermal properties of the materials affect the accuracy of heat loss calculations in the energy balance, leading to increased heating or cooling costs for buildings.

**Figure 5:**
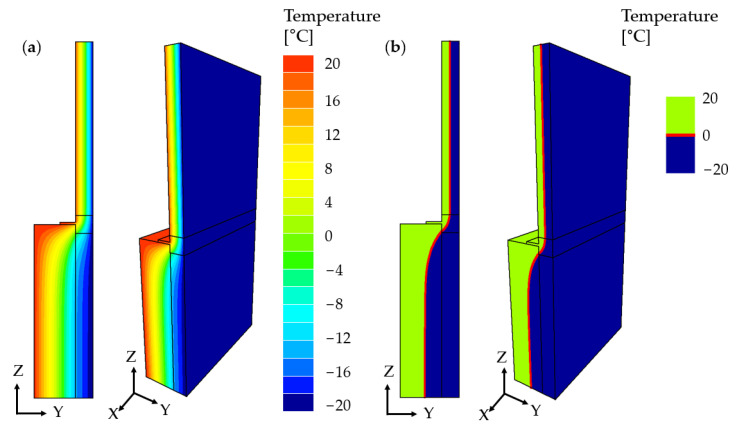
Numerical simulation results: (**a**) temperature distribution in the cross-section, (**b**) position of the 0 °C isotherm.

The results of the numerical simulations were analyzed to determine the temperature distribution in the cross-sections of the wall variants. Figure 5 illustrates the temperature distribution and the position of the 0 °C isotherm for mineral wool used as the thermal insulating layer.

For all the analyzed external wall variants, a similar temperature distribution was observed, with the 0 °C isotherm partially running through the load-bearing layer in each case. This indicates that during winter, with an external temperature of −20 °C, there is a risk of water particles in the pores and joints of the load-bearing layer freezing. In the case of damp walls, cyclic freezing and thawing during winter can lead to damage not only to the masonry but also to any installations within the wall, potentially resulting in damage to the entire building structure. In such instances, increasing the thickness of the thermal insulation layer is necessary to move the 0 °C isotherm into the thermal insulation layer. 

To examine the moisture properties of the analyzed external wall variants, the risk of mold growth on the internal surface of the wall was assessed. For this purpose, the TRISCO program was used to determine the temperature on the internal surface of the wall and to calculate the temperature factor *f_Rsi_* (Table 9). Simulations were conducted for walls with a thermal insulation layer thickness of 0.10 m.

Condition (4) was met in all variants of the analyzed external walls, indicating that the calculated values of the temperature factors *f_Rsi_* are greater than the critical value of the temperature factor *f_Rsi_*_(*crit*)_, which is 0.72. Therefore, it can be concluded that the proposed external wall configuration does not pose a risk of surface condensation or mold growth.

The impact of the thermal conductivity coefficient of the thermal insulation material on the size of the linear thermal bridge at the junction of the window and the external wall was also analyzed. The values of the linear heat transmittance coefficient are provided in Table 10. 

The value of the linear heat transmittance coefficient *Ψ* exhibited an average increase of 4% when using the measured thermal conductivity values for the thermal insulation layer, compared to instances where the declared values of the thermal conductivity coefficient *λ* were used. In the case of a rigid foamed composite, the increase was as high as 13%. Although each case met Condition (7) and, according to The Passive House Institute’s guidelines [35], can be considered “free from linear thermal bridges”, it remains necessary to use measured and therefore corrected to the actual storage conditions values, rather than declared thermal conductivity values for thermal insulation materials in heat loss calculations due to their impact on the building’s energy balance. 

The ability of a material to conduct heat is directly related to its structure. Materials with low thermal conductivity, such as those with a high proportion of air pockets, are generally better insulators. Typically, materials with lower densities offer better insulating properties because they contain more air, which is a poor conductor of heat. However, when materials absorb moisture, their insulating effectiveness can diminish, as water conducts heat more efficiently than air. Additionally, materials that degrade or compress over time can lose their insulating properties. By understanding and optimizing these structural characteristics, it is possible to develop insulation materials that provide superior energy efficiency and durability.

Future experimental research on the changes in insulation material properties will focus on the following areas:

- further analysis of the impact of environmental conditions and storage conditions on samples (e.g., extended storage periods, storage during the spring and summer seasons),
- determining the effect of insulation material thickness on thermal conductivity,
- investigating the hygroscopic properties of selected insulation materials,
- conducting analyses on insulation materials derived from recycling.

Further numerical analysis and analytical calculations should include:

- assessing the risk of interstitial condensation in external walls,
- evaluating the potential use of artificial intelligence tools in assessing thermal and moisture properties of insulation materials.

## 4. Conclusions

The study conducted a comprehensive examination and analysis of the material properties of various types of thermal insulation materials, including well-established products like polystyrene and mineral wool, as well as modern materials such as composite boards. The experimental studies and numerical analysis of heat flow through the external wall yielded the following conclusions:

- the analysis and testing of thermal insulation materials showed that improper storage of samples leads to a deterioration in thermal properties,
- inadequate storage resulted in a 4% increase in the thermal conductivity coefficient for standard thermal insulation materials and a 19% increase for the composite material,
- the measurements of thermal insulation materials with a heterogeneous structure reveal greater uncertainty,
- the lack of knowledge regarding the actual thermal properties of materials, and consequently, inappropriate selection of insulation layer thicknesses, affects the basic thermal performance of external walls; this, in particular, leads to increased heat loss through the largest structural elements of buildings, directly resulting in higher heating costs and indirectly in increased greenhouse gas emissions,
- each analysed configuration of external wall layers should be assessed during the design phase for the risk of surface condensation and the position of the wall frost line,
- the thermal parameters of the thermal insulation layer influence the value of the linear heat transmittance coefficient *Ψ* resulting from window installation.

A notable contribution of this work for researchers in the field of building energy efficiency is its focus on the often-overlooked aspect of insulation material storage, particularly for materials susceptible to moisture absorption, such as mineral wool. The study provides valuable data on the thermal conductivity of these materials both post-manufacture and after storage under warehouse and outdoor conditions. The numerical analyses demonstrate a significant effect of insulation materials’ thermal properties on temperature distribution within external walls with windows, which directly influences the overall thermal performance of buildings. 

The results from both our experimental and numerical analyses will not only benefit practitioners and designers but also provide key insights for scientists focused on improving building thermal efficiency. The findings have practical implications for the construction industry by offering guidelines for choosing the most effective insulation materials. This can help bridge the gap between research and real-world applications, thereby supporting the adoption of energy-saving technologies.

Looking forward, future research could explore opportunities for collaborations between universities, research institutions, and industry. Such partnerships could drive the development and widespread implementation of innovative solutions aimed at enhancing building thermal efficiency and accelerating the adoption of energy-efficient technologies. Additionally, the research outcomes may serve as valuable resources for educational purposes and design guides, supporting architects, designers, contractors, and investors in their decision-making processes.

## Figures and Tables

**Table 1:** Declared thermal conductivity values *λ_D_* for the analyzed materials.

Insulation Material	Heat Transfer Coefficient *λ_D_* [W/(m·K)]
XPS extruded polystyrene	0.034
PIR polyisocyanurate foam	0.022
Mineral wool	0.035
EPS expanded polystyrene	0.032
Rigid foamed composite	0.020

**Table 2:** Average thermal conductivity values *λ_avg_* for the control samples (Group 1).

Insulation Material	Mass [kg]	Density [kg/m^3^]	Test Time [min]	Average Test Time [min]	Coefficient *λ* [W/(m·K)]	Average Coefficient *λ_avg_* [W/(m·K)]
XPS extruded polystyrene	0.152	17.1	90	76	0.03401	0.03422
			59		0.03454	
			78		003411	
PIR polyisocyanurate foam	0.278	30.5	213	482	0.02201	0.02230
			696		0.02282	
			536		0.02206	
Mineral wool	0.696	77.7	91	108	0.03467	0.03532
			107		0.03524	
			127		0.03606	
EPS expanded polystyrene	0.200	22.0	72	75	0.03271	0.03233
			70		0.03221	
			84		0.03208	
Rigid foamed composite	0.554	61.4	1145	700	0.02272	0.02229
			242		0.02202	
			714		0.02213	

**Table 3:** Average thermal conductivity values *λ_avg_* for samples stored inside an unheated storage room (Group 2).

Insulation Material	Mass [kg]	Density [kg/m^3^]	Test Time [min]	Average Test Time [min]	Coefficient *λ* [W/(m·K)]	Average Coefficient *λ_avg_* [W/(m·K)]
XPS extruded polystyrene	0.152	17.1	67	78	0.03405	0.03408
			100		0.03404	
			66		003414	
PIR polyisocyanurate foam	0.286	31.3	267	458	0.02282	0.02222
			653		0.02139	
			454		0.02244	
Mineral wool	0.745	83.3	68	94	0.03608	0.03524
			116		003516	
			97		0.03449	
EPS expanded polystyrene	0.202	22.5	92	89	0.03217	0.03247
			95		0.03253	
			80		0.03271	
Rigid foamed composite	0.554	61.4	1150	1173	0.02216	0.02214
			1420		0.02247	
			949		0.02178	

**Table 4:** Average thermal conductivity values *λ_avg_* for samples stored outdoors (Group 3).

Insulation Material	Mass [kg]	Density [kg/m^3^]	Test Time [min]	Average Test Time [min]	Coefficient *λ* [W/(m·K)]	Average Coefficient *λ_avg_* [W/(m·K)]
XPS extruded polystyrene	0.150	17.0.	98	95	0.03544	0.03541
			123		0.03531	
			65		0.03548	
PIR polyisocyanurate foam	0.281	30.7	142	701	0.02235	0.02297
			824		0.02367	
			1136		0.02289	
Mineral wool	0.719	80.2	107	133	0.03649	0.03688
			110		0.03737	
			183		0.03677	
EPS expanded polystyrene	0.198	22.1	85	106	0.03361	0.03317
			107		0.03296	
			127		0.03294	
Rigid foamed composite	0.574	63.6	204	224	0.02366	0.02380
			213		0.02371	
			255		0.02402	

**Table 5:** Comparison of thermal conductivity values of thermal insulation materials.

Insulation Material	Average Coefficient *λ_avg_* [W/(m·K)]				
	Declared Values	Group 1 Samples (Control)	Group 2 Samples	Group 3 Samples	Relative Error
XPS extruded polystyrene	0.034	0.03422	0.03408	0.03541	4.1
PIR polyisocyanurate foam	0.022	0.02230	0.02222	0.02297	4.4
Mineral wool	0.035	0.03532	0.03524	0.03688	5.4
EPS expanded polystyrene	0.032	0.03233	0.03247	0.03317	3.7
Rigid foamed composite	0.020	0.02229	0.02214	0.02380	19.0

**Table 6:** Statistical data for the thermal conductivity tests.

Insulation Material	Standard Deviation s_d_ [W/(m·K)]					
	Group 1 Samples (Control)		Group 2 Samples		Group 3 Samples	
	Average Coefficient *λ_avg_*	Standard Deviation s_d_	Average Coefficient *λ_avg_*	Standard Deviation s_d_	Average Coefficient *λ_avg_*	Standard Deviation s_d_
XPS extruded polystyrene	0.03422	0.00028	0.03408	0.00006	0.03541	0.00009
PIR polyisocyanurate foam	0.02230	0.00045	0.02222	0.00074	0.02297	0.00066
Mineral wool	0.03532	0.00070	0.03524	0.00080	0.03688	0.00045
EPS expanded polystyrene	0.03233	0.00033	0.03247	0.00027	0.03317	0.00038
Rigid foamed composite	0.02229	0.00038	0.02214	0.00035	0.02380	0.00020

**Table 7:** Corrected heat transfer coefficient values *U_c_* for an insulation layer thickness of 0.10 m.

Insulation Material	Thermal Conductivity Coefficients *λ* W/(m·K)		Heat Transfer Coefficient *U_C_* W/(m^2^·K)
XPS extruded polystyrene	Declared value *λ_D_*	0.034	0.230
	Intended value *λ_avg_*	0.035	0.234
PIR polyisocyanurate foam	Declared value *λ_D_*	0.022	0.178
	Intended value *λ_avg_*	0.023	0.183
Mineral wool	Declared value *λ_D_*	0.035	0.234
	Intended value *λ_avg_*	0.037	0.241
EPS expanded polystyrene	Declared value *λ_D_*	0.032	0.222
	Intended value *λ_avg_*	0.033	0.226
Rigid foamed composite	Declared value *λ_D_*	0.020	0.168
	Intended value *λ_avg_*	0.024	0.188

**Table 8:** Corrected heat transfer coefficient values *Uc* meeting the regulatory requirements.

Insulation Material	Thermal Conductivity Coefficients *λ* W/(m·K)		Insulation Layer Thickness [m]	Heat Transfer Coefficient *U_c_* W/(m^2^·K)
XPS extruded polystyrene	Declared value *λ_D_*	0.034	0.13	0.198
	Intended value *λ_avg_*	0.035	0.14	0.192
PIR polyisocyanurate foam	Declared value *λ_D_*	0.022	-	0.178
	Intended value *λ_avg_*	0.023	-	0.183
Mineral wool	Declared value *λ_D_*	0.035	0.14	0.192
	Intended value *λ_avg_*	0.037	0.15	0.191
EPS expanded polystyrene	Declared value *λ_D_*	0.032	0.13	0.191
	Intended value *λ_avg_*	0.033	0.13	0.194
Rigid foamed composite	Declared value *λ_D_*	0.020	-	0.168
	Intended value *λ_avg_*	0.024	-	0.188

**Table 9:** Values of the temperature factor *f_Rsi_* calculated using the TRISCO program.

Insulation Material	Thermal Conductivity Coefficients *λ* W/(m·K)		Wall Surface Temperature [°C]	Temperature Factor *f_Rsi_* [−]
XPS extruded polystyrene	Declared value *λ_D_*	0.034	18.87	0.972
	Intended value *λ_avg_*	0.035	18.83	0.971
PIR polyisocyanurate foam	Declared value λ_D_	0.022	19.16	0.979
	Intended value λ_avg_	0.023	19.11	0.978
Mineral wool	Declared value λ_D_	0.035	18.85	0.971
	Intended value *λ_avg_*	0.037	18.79	0.970
EPS expanded polystyrene	Declared value λ_D_	0.032	18.83	0.971
	Intended value λ_avg_	0.033	18.81	0.970
Rigid foamed composite	Declared value λ_D_	0.020	19.22	0.981
	Intended value *λ_avg_*	0.024	19.06	0.976

**Table 10:** Values of the linear heat transmittance coefficient *Ψ*.

Insulation Material	Thermal Conductivity Coefficients λ W/(m·K)		Linear Heat Transmittance Coefficient *Ψ* [W/(m·K)]
XPS extruded polystyrene	Declared value λ_D_	0.034	0.0082298
	Intended value *λ_avg_*	0.035	0.0086556
PIR polyisocyanurate foam	Declared value λ_D_	0.022	0.0054062
	Intended value λ_avg_	0.023	0.0056367
Mineral wool	Declared value λ_D_	0.035	0.0086556
	Intended value *λ_avg_*	0.037	0.0089941
EPS expanded polystyrene	Declared value λ_D_	0.032	0.0078780
	Intended value λ_avg_	0.033	0.0081364
Rigid foamed composite	Declared value λ_D_	0.020	0.0050722
	Intended value *λ_avg_*	0.024	0.0057369

## Data Availability

The data presented in this study are available on request from the corresponding author.

## Supplementary Materials

The following supporting information can be downloaded at: https://www.mdpi.com/article/10.3390/ma17194718/s1, File S1 “sample XPS_control_group.xlsx”.
